# Correlation between large rearrangements and patient phenotypes in *NF1* deletion syndrome: an update and review

**DOI:** 10.1186/s12920-024-01843-5

**Published:** 2024-03-06

**Authors:** Laurence Pacot, Milind Girish, Samantha Knight, Gill Spurlock, Vinod Varghese, Manuela Ye, Nick Thomas, Eric Pasmant, Meena Upadhyaya

**Affiliations:** 1 Fédération de Génétique et Médecine Génomique, Hôpital Cochin, DMU BioPhyGen, AP-HP, Centre-Université Paris Cité, Paris, France; 2 Institut Cochin, Inserm U1016, CNRS UMR8104, Université Paris Cité, CARPEM, Paris, France; 3 School of Clinical Medicine, University of Cambridge, Cambridge, UK; 4 Division of Cancer and Genetics, Institute of Medical Genetics, Cardiff University, Heath Park, CF14 4XN Cardiff, UK; 5 Cardiff University, Cardiff, Great Britain; 6 All Wales Medical Genomics Service, Cardiff, Great Britain

**Keywords:** Neurofibromatosis type 1, NF1, Deletion, CNV, Genotype-phenotype correlation

## Abstract

About 5–10% of neurofibromatosis type 1 (NF1) patients exhibit large genomic germline deletions that remove the *NF1* gene and its flanking regions. The most frequent *NF1* large deletion is 1.4 Mb, resulting from homologous recombination between two low copy repeats. This “type-1” deletion is associated with a severe clinical phenotype in NF1 patients, with several phenotypic manifestations including learning disability, a much earlier development of cutaneous neurofibromas, an increased tumour risk, and cardiovascular malformations. *NF1* adjacent co-deleted genes could act as modifier loci for the specific clinical manifestations observed in deleted NF1 patients. Furthermore, other genetic modifiers (such as CNVs) not located at the *NF1* locus could also modulate the phenotype observed in patients with large deletions. In this study, we analysed 22 *NF1* deletion patients by genome-wide array-CGH with the aim (1) to correlate deletion length to observed phenotypic features and their severity in *NF1* deletion syndrome, and (2) to identify whether the deletion phenotype could also be modulated by copy number variations elsewhere in the genome. We then review the role of co-deleted genes in the 1.4 Mb interval of type-1 deletions, and their possible implication in the main clinical features observed in this high-risk group of NF1 patients.

**Supplementary Information:**

The online version contains supplementary material available at 10.1186/s12920-024-01843-5.

## Introduction

Neurofibromatosis constitutes an autosomal inherited condition associated with tumour development of the nervous system. NF1 (neurofibromatosis type 1) forms the most common type of this disorder, with an estimated incidence of 1:3000 live births worldwide and is caused by heterozygous loss-of-function variants of the *NF1* gene at 17q11.2. The commonest NF1-associated tumours are benign peripheral nerve tumours that may be cutaneous, subcutaneous, or plexiform neurofibromas.

**Fig. 1:**
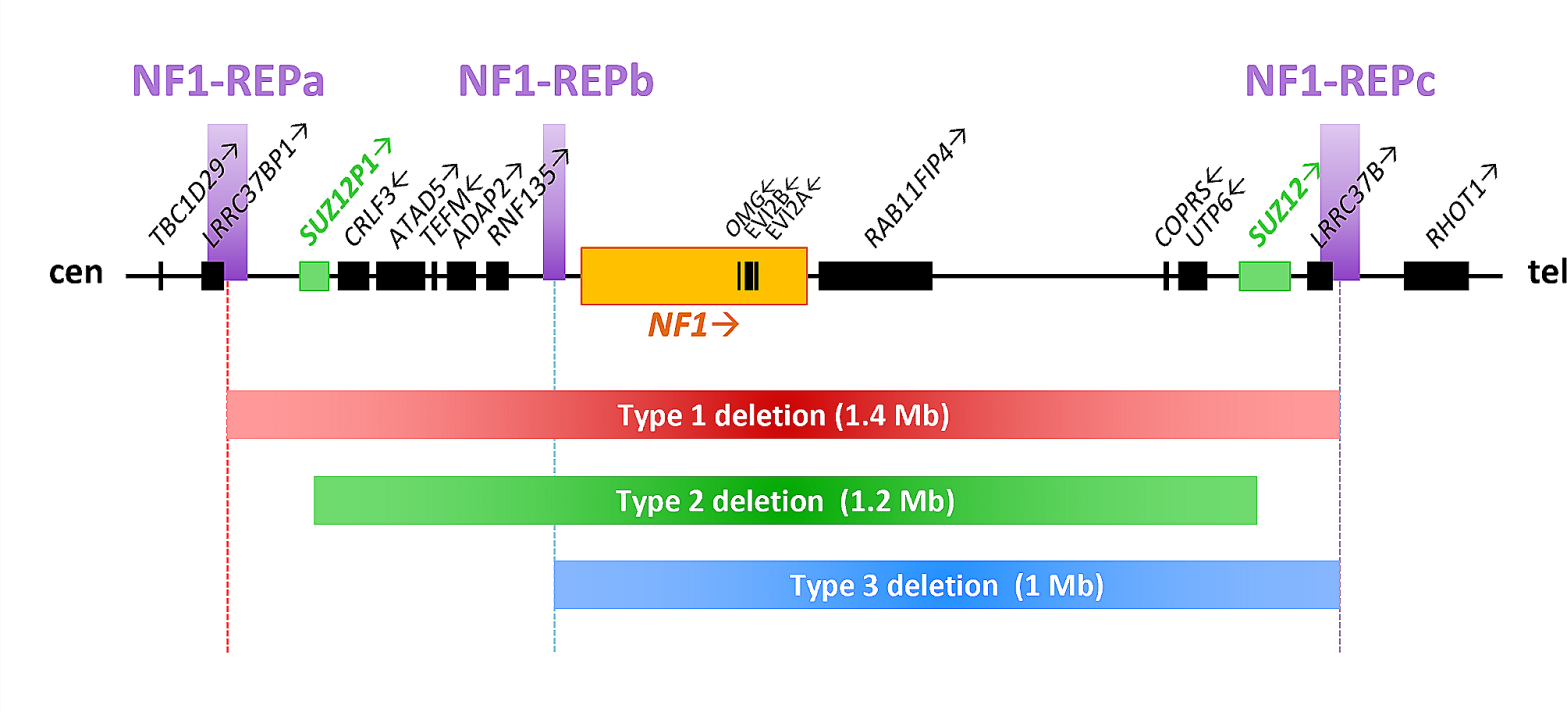
Illustration of the genomic region harboring the *NF1* and adjacent genes. Red, green, and blue intervals represent the deletion extent in the recurrently observed type-1, type-2, and type-3 *NF1* deletions respectively. *NF1* is depicted in orange; *SUZ12* and its pseudogene *SUZ12P1* in green. Purple intervals indicate low-copy repeats (LCRs) implicated in non-allelic homologous recombination (NAHR) at the origin of type-1 and type-3 deletions. Arrows adjacent to gene symbols denote transcriptional orientation. Cen and tel refer to centromeric and telomeric direction, respectively

About 4–10% of NF1 patients carry large heterozygous genomic germline deletions that remove the *NF1* tumour suppressor gene and its flanking regions [1]. Within this subsidiary, four delineated ‘types’ of *NF1* deletion are observed and classified based on the length of deletion (Fig. 1) [2]. Type-1 deletions of 1.4 Mb are far more common than type-2 or type-3 deletions that span 1.2 Mb and 1.0 Mb respectively, whilst the other atypical deletions are more diverse [3]. Type-1 deletions mostly result from meiotic NAHR between *NF1-REPa* and *NF1-REPc* and encompass a 1.4 Mb region including 14 protein coding genes and 4 microRNA genes. Type-2 deletions are mediated by NAHR between *SUZ12* and its pseudogene *SUZ12P1*, often occurring post-zygotically and leading to mosaicism [4, 5]. Type-3 deletions are less frequent (about 1–4% of all *NF1* deletions) and are caused by NAHR between *NF1-REPb* and *NF1-REPc* [6, 7]. Non homologous end joining (NHEJ) or replication based mechanisms have been implicated in atypical deletions [3, 8–11].

Characterisation of the *NF1* deletion syndrome is clinically of paramount importance due to the comparatively more severe clinical phenotype *versus* patients with *NF1* point mutations [12]. A study of 29 patients [13] with type-1 *NF1* deletions highlighted exacerbated features such as much earlier onset of cutaneous neurofibromas, dysmorphic facial features, large hands and feet, scoliosis, developmental delay, learning disabilities, accumulation of high tumour burden, cardiovascular malformations and a much higher risk of developing a malignant peripheral nerve sheath tumour (MPNST), an aggressive cancer associated with a poor prognosis [14]. MPNST lifetime risk across all NF1 patients is estimated to be 8–13%, but this estimation doubles to 16–26% in *NF1* deletion patients [15]. Additionally, in direct contrast to the shortened stature frequently observed in patients exhibiting intragenic *NF1* mutations, *NF1* deletion patients more typically exhibit childhood overgrowth and tall stature in adults [16]. *NF1* deletion patients significantly more often presented with symptomatic spinal neurofibromas, cardiovascular and skeletal abnormalities, learning disabilities, dysmorphism, and malignancies when compared to a “classic NF1” cohort, in a cohort of 126 *NF1* deletions patients [1].

Undoubtedly, this severe clinical presentation in *NF1* deletion patients could be correlated to the co-deleted genes in the region. Moreover, beyond considering these genes in isolation, another plausible mechanism could be that haploinsufficiency of these genes may synergize with each other, or with the *NF1* loss, to induce the specific *NF1* deletion syndrome (what may be called a contiguous gene syndrome). Additionally, whilst NF1 exhibits complete penetrance, its expressivity varies dramatically. Thus, establishing genotype to phenotype correlations could extract significant benefit in guiding pre-emptive clinical support and management of such an intractable disorder, particularly in the proportionally severe subset of *NF1* deletion syndrome.

In this study, we analysed 22 *NF1* deletion patients by genome-wide array-CGH with the aim (1) to correlate deletion length to observed phenotypic features and their severity in *NF1* deletion syndrome, and (2) to identify whether the deletion phenotype could also be modulated by modifier copy number variations (CNVs) elsewhere in the genome. We also propose a review of the literature to suggest candidate modifier genes for the variable expressivity of NF1, among the genes located in the recurrent deletion interval.

## Materials and methods

### Patients and DNA analyses

We have analysed 22 unrelated *NF1* deletion patients. DNA from these patients was tested using Human 1 M-Duo SNP chips (patients) and Human Omni1-Quad SNP chips (parents and sibling), respectively, according to manufacturer’s guidelines (Illumina, San Diego, CA). Briefly, for each sample ~ 200ng DNA were denatured, amplified, enzymatically fragmented, and hybridized to the BeadChips in a hybridization oven (Illumina) at 48˚C for 16-24 h. The BeadChips were washed according to the manufacturer’s protocol and the hybridized DNA subjected to primer extension with labelled nucleotides prior to detection using fluorescent antibodies. Data were processed using GenomeStudioV2009.2 (Illumina) and analysed using Nexus Discovery Edition v6.1 (BioDiscovery, Hawthorne, CA). All Nexus plots were inspected visually to verify calls made, identify uncalled events, and to exclude likely false positives. To exclude common germline CNVs, the Database of Genomic Variants (DGV), a comprehensive catalogue of structural variation in control data, was used. Copy number changes that encompassed changes noted in the DGV or identified in a clinically normal parent were excluded from further analysis. Regions of copy neutral loss of heterozygosity (cnLOH) were recorded only if > 5 Mb in size. This minimised the reporting of common cnLOH events that occurred in controls but would not rule out events arising through consanguinity. All genomic coordinates are given with reference to the GRCh36, hg18 assembly. Bioinformatic analysis of SNPs was also carried out. A correlation between SNPs and a specific clinical feature was explored.

### Statistical analysis

Assessment was undertaken through the tabulation of summary statistics for values under consideration. Median (range) and frequencies (percentages) for the association between two covariates were used for the description of the relationship. The significance level for all statistical tests was 0.05. Note that the primary aim to identify modifying loci that interact with partial *NF1*-loss to induce a more severe phenotype in *NF1* deletion syndrome uses multiple comparisons, and multiplicity problems could appear in this analysis. Multiplicity may inflate the type I error (α) and the probability of finding a significant association just by chance, a false-positive conclusion. Each test has a 5% chance of a false positive result when there is no real association (a type I error) so if the analysis has multiple comparisons the probability of at least one false positive result is very much greater than 5%. Consequently, the significance level for all statistical tests could be adjusted. A simple and intuitive multiplicity adjustment is the Bonferroni method, which requires that the p-value for each comparison be less than or equal to 0.05 divided by the total number of comparisons. In this analysis, the primary aim performs 112 comparisons, then the Bonferroni adjustment method would require a p-value less than or equal to 0.00045. The disadvantage of multiplicity adjustment methods is that they can be quite conservative if there are many comparisons and could increase β thereby reducing statistical power. The analysis was performed using the statistical software STATA11.

## Results

### Clinical data

We have analysed 22 unrelated *NF1* deletion patients, including 18 patients with a type-1 deletion, three patients with a type-2 deletion, and one patient with an atypical > 5.5 Mb deletion. Clinical details were also recorded from these 22 patients.

Available clinical information for the 22 patients and comparison with the general NF1 population are summarised in Table 1; however, it should be noted that not all clinical information was available.

**Table 1:** Clinical and genetic data for the 22 patients included in the study, compared to the general NF1 population

Feature	Number of patients presenting the phenotype	Frequency in the general NF1 population^1^	p-value^4^
Plexiform Neurofibroma	11/19 (58%)	120/648 (18.5%)^2^	0.00021
Lisch Nodules	10/14 (71%)	729/1237 (58.9%)	0.42
Macrocephaly	10/15 (67%)	239/704 (33.9%)	0.012
Learning Difficulties	16/18 (89%)	190/424 (44.8%)	0.00035
Scoliosis	5/19 (26%)	51/236 (21.6%)^3^	0.58
Other Malignancies	4/21 (19%)	18/523 (3.4%)	0.0077

^1^ Data from Koczkowska et al. [17].

^2^ Major external plexiform neurofibromas in individuals > 8 years old

^3^ In individuals > 18 years old

^4^ Two-tailed Fisher’s exact test performed with the R stats package in RStudio v4.2.2

Analysis of five nuclear families with *NF1* deletion child and unaffected parents failed to reveal additional novel CNVs in the proband. We then looked for CNVs in the combined 22 deletion patients. No significant findings were observed.

### Association between NF1 deletion and patient features

Using *NF1* length as continuous variable to assess its relationship with patient features, patients with learning difficulties were more likely to show higher *NF1* deletion length at significant level *p* = 0.025 (Table 2, Mann-Whitney test). However, the number of patients was too small to draw a reliable conclusion. The length was not associated with any of other observed features.

‘Patient 2’ was not included in the analysis, as he exhibited an atypical deletion > 5.5 Mb far larger than the deletions observed in 18 patients with a type-1 deletion and 3 patients with a type-2 deletion.

**Table 2:** Correlation between clinical features and deletion length (excluding Patient 2 with deletion > 5.5 Mb)

			Length	p-value^1^
			Median (range)	
Overall Severity of NF	Mild/moderate	*N* = 9	1,339,470 (1,136,399–1,472,314)	0.1356
	Severe	*N* = 12	1,383,592 (1,172,942–1,501,344)	
Plexiform neurofibromas	No	*N* = 7	1,361,336 (1,172,942–1,415,978)	0.2576
	Yes	*N* = 11	1,378,007 (1,170,165–1,501,344)	
Lisch Nodules	No	*N* = 4	1,349,299 (1,308,064–1,446,618)	0.4404
	Yes	*N* = 9	1,376,701 (1,136,399–1,472,314)	
Macrocephaly	No	*N* = 5	1,376,701 (1,308,064–1,446,618)	0.7389
	Yes	*N* = 9	1,375,509 (1,170,165–1,415,978)	
Learning Difficulties	No	*N* = 2	1,171,554 (1,170,165–1,172,942)	**0.0253**
	Yes	*N* = 15	1,376,701 (1,308,064–1,501,344)	
Scoliosis	No	*N* = 13	1,378,007 (1,170,165–1,501,344)	0.8825
	Yes	*N* = 5	1,376,701 (1,339,470–1,437,879)	
Other Malignancies	No	*N* = 16	1,376,758 (1,170,165–1,501,344)	0.3447
	Yes	*N* = 4	1,407,290 (1,361,336–1,472,314)	

^1^ Mann-Whitney test

Comparison of total *NF1* deletion size > 1.2 Mb between each patient feature using Fisher’s exact test concluded that patients with learning difficulties showed higher percentage of *NF1* deletion size > 1.2 Mb (100%) than patients without (0%) (*p* = 0.007, Fisher’s exact test; not significant after Bonferroni correction). Significance was not shown in any other features (Supplementary Tables S1 and S2). Same comparisons were carried out for total *NF1* deletion size > 1.35 Mb between each patient feature, the results did not show any difference between patients with and without *NF1* deletion size > 1.35 Mb, in relationship to the patient features (Supplementary Tables S3 and S4). In addition, there was no association shown in the comparisons for total *NF1* deletion size > 1.40 Mb between each patient features (Supplementary Tables S5 and S6).

### Association between gene loss in NF1 deletion and patient features

To assess the association between gene loss in *NF1* deletion and patient features, results concluded that patients with learning difficulties showed higher number of genes loss in *NF1* deletion than patients without learning difficulties at p-value equal to 0.0087 (Mann-Whitney test; not significant after Bonferroni correction) (Table 3). Please also note that the number in the ‘learning difficulties’ symptom is extremely small.

**Table 3:** Correlation between clinical features and number of genes lost in *NF1* deletion (excluding Patient 2 with deletion > 5.5 Mb)

			Genes lost in *NF1* deletion	
			Median (range)	p-value^1^
Overall Severity of NF	Mild/moderate	*N* = 9	33 (25–37)	0.3435
	Severe	*N* = 12	33 (26–37)	
Plexiform neurofibromas	No	*N* = 7	33 (26–33)	0.0632
	Yes	*N* = 11	33 (26–37)	
Lisch Nodules	No	*N* = 4	33 (32–36)	0.7915
	Yes	*N* = 9	33 (25–37)	
Macrocephaly	No	*N* = 5	33 (32–36)	0.4515
	Yes	*N* = 9	33 (26–33)	
Learning Difficulties	No	*N* = 2	26 (26–26)	**0.0087**
	Yes	*N* = 15	33 (32–37)	
Scoliosis	No	*N* = 13	33 (26–37)	0.5146
	Yes	*N* = 5	33 (32–33)	
Other Malignancies	No	*N* = 16	33 (26–37)	0.8725
	Yes	*N* = 4	33 (32–37)	

^1^ Mann-Whitney test

### Association between genes loss in NF1 and other sites and patient features

Comparison of number of genes loss in *NF1* deletion, other non-DGV, non-inherited, and other gains between each patient feature were carried out using non-parametric Mann-Whitney test. See Tables 4, 5 and 6 for details. Results concluded that patients with learning difficulties showed higher number of genes loss in *NF1* region and other sites (statistically significant association, p-value < 0.05; not significant after Bonferroni correction). However, the number of patients without learning difficulties was, again, too small (*n* = 2) to draw a reliable conclusion. A borderline significance was observed between the loss of several genes and plexiform neurofibromas (Table 5), when Patient 2 excluded. This observation must be confirmed by a larger independent study.

**Table 4:** Correlation between clinical features and number of genes lost considering (1) genes in *NF1* deletion, (2) ‘genes in other non-DGV, non-inherited losses’, and (3) ‘genes in other gains’

			Median of genes lost (range)	p-value^1^
Overall Severity of NF	Mild/moderate	*N* = 9	34 (26–372)	0.9424
	Severe	*N* = 12	33 (26–37)	
Plexiform neurofibromas	No	*N* = 7	33 (26–35)	0.0882
	Yes	*N* = 11	34 (26–372)	
Lisch Nodules	No	*N* = 4	34 (32–36)	0.3820
	Yes	*N* = 9	33 (27–37)	
Macrocephaly	No	*N* = 5	34 (32–36)	0.4073
	Yes	*N* = 9	33 (26–35)	
Learning Difficulties	No	*N* = 2	26 (26–26)	**0.0217**
	Yes	*N* = 15	33 (32–372)	
Scoliosis	No	*N* = 13	34 (26–53)	0.3384
	Yes	*N* = 5	33 (32–34)	
Other Malignancies	No	*N* = 16	34 (26–372)	0.5636
	Yes	*N* = 4	33 (32–37)	

^1^ Mann-Whitney test

**Table 5:** Correlation between clinical features and number of genes lost (excluding Patient 2 with deletion > 5.5 Mb) and considering genes in *NF1* deletion + ‘genes in other non-DGV, non-inherited losses’ + ‘genes in other gains’. Patients with plexiform neurofibromas showed slightly high number of genes loss in *NF1* region and other sites (borderline significant association, p-value = 0.0488)

			Median of genes lost (range)	p-value^1^
Overall Severity of NF	Mild/moderate	*N* = 9	34 (25–227)	0.7462
	Severe	*N* = 12	33.5 (26–39)	
Plexiform neurofibromas	No	*N* = 7	33 (26–35)	**0.0488**
	Yes	*N* = 11	34 (26–227)	
Lisch Nodules	No	*N* = 4	34 (32–36)	0.6936
	Yes	*N* = 9	33 (25–39)	
Macrocephaly	No	*N* = 5	34 (32–39)	0.1332
	Yes	*N* = 9	33 (26–35)	
Learning Difficulties	No	*N* = 2	26 (26–26)	**0.0230**
	Yes	*N* = 15	34 (32–227)	
Scoliosis	No	*N* = 13	34 (26–37)	0.9202
	Yes	*N* = 5	33 (32–39)	
Other Malignancies	No	*N* = 16	34 (26–227)	0.6664
	Yes	*N* = 4	35 (32–39)	

^1^ Mann-Whitney test

**Table 6:** Correlation between clinical features and number of genes lost (excluding Patient 2 with deletion > 5.5 Mb) and considering genes in *NF1* deletion + ‘genes in other non-DGV, non-inherited losses’

			Median of genes lost (range)	p-value^1^
Overall Severity of NF	Mild/moderate	*N* = 9	33 (25–227)	0.8820
	Severe	*N* = 12	33 (26–37)	
Plexiform neurofibromas	No	*N* = 7	33 (26–34)	0.0711
	Yes	*N* = 11	33 (26–227)	
Lisch Nodules	No	*N* = 4	33.5 (32–36)	0.5688
	Yes	*N* = 9	33 (25–37)	
Macrocephaly	No	*N* = 5	33 (32–36)	0.3359
	Yes	*N* = 9	33 (26–34)	
Learning Difficulties	No	*N* = 2	26 (26–26)	**0.0152**
	Yes	*N* = 15	33 (32–227)	
Scoliosis	No	*N* = 13	33 (26–37)	0.3979
	Yes	*N* = 5	33 (32–33)	
Other Malignancies	No	*N* = 16	33 (26–227)	0.8038
	Yes	*N* = 4	33 (32–37)	

^1^ Mann-Whitney test

### Candidate modifier genes localized in the 1.4 mb type-1 deletion region and their putative role in clinical expressivity of NF1

The 1.4 Mb locus encompassed by *NF1* type-1 deletions comprises 13 protein coding genes, and 5 microRNA genes. We summarize here what is known about these genes, and how they might be implicated in the severe phenotype evidenced in this group of patients (Table 7).

#### SUZ12

*SUZ12* is located approximately 40 kb telomeric to the *NF1* gene. Both type-1 and type-3 deletions encompass the gene, and recurrent recombination events with its pseudogene, *SUZ12P1*, is responsible for type-2 deletions (Fig. 1). Fusion transcript analysis in type-2 deletion patients identified chimeric sequences predicted to contain premature stop codons, presumably coding for truncated proteins, or no protein at all [18].

SUZ12 is a 739 amino-acid protein with ubiquitous expression. As a core subunit of the Polycomb Repressive Complex 2 (PRC2) together with embryonic ectoderm development (EED), the histone methyltransferase enhancer of zeste homologue 1/2 (EZH1/2), and with RB binding protein 4 or 7 (RBBP4 or RBBP7), the PRC2 catalyses the mono-, di- and tri-methylation of histone H3 at lysine 27, implicated in the maintenance of transcriptional repression of several target genes in a cell-type and differentiation-stage specific manner [19]. In vivo knock-out models have shown that PRC2 core proteins (EED, EZH1/EZH2, and SUZ12) are essential for embryonic development, as illustrated by embryonic lethality in *Suz12*^*−/−*^ mouse around gastrulation [20].

In human, heterozygous loss-of-function variants affecting PRC2 core proteins genes are responsible for overgrowth with intellectual disability disorders (OGID) [21]. Weaver syndrome (WS) is caused by mutations in *EZH2* (Enhancer of Zeste homolog 2) [22] and is characterized by pre- and/or postnatal overgrowth, macrocephaly, advanced bone age, distinctive craniofacial features, and a variable degree of intellectual disability. Musculoskeletal abnormalities are also frequently observed. Some patients develop childhood tumours [23–25]. Cohen-Gibson syndrome (COGIS) was described later, involving mutations in *EED* (Embryonic Ectoderm Development) [26], and can be distinguished from Weaver syndrome by more prevalent cryptorchidism, cervical spine abnormalities, and cardiac abnormalities [27]. The first case report for an heterozygous *SUZ12* pathogenic variant in human was described in 2017 in an 11-year-old girl [28]. The variant was inherited from her father, who showed mosaicism for the mutation and had a milder clinical presentation. Later reports by the same group led the condition to become Imagawa-Matsumoto syndrome (IMMAS) [29]. Affected individuals develop malformations and disabilities generally milder than those observed in other OGID due to PRC2 mutations, with the exception of a more prominent increase in postnatal head circumference, and the presence in some but not all IMMAS patients of hypertrichosis, a condition never described in WS and COGIS [29]. A recently published patient with a 1.4 Mb deletion encompassing *SUZ12*, but not *NF1*, confirms the consequences of *SUZ12* haploinsufficiency: large hands and feet, hyperlaxity, intellectual disability, macrocephaly, dysmorphism, and postnatal overgrowth [30]. Altogether, *SUZ12* haploinsufficiency can more certainly be at least in part responsible for the learning disabilities and the overgrowth phenotype observed in some *NF1*-deleted patients.

Several studies have shown the role of PRC2 in central nervous system (CNS) development. In vitro or in vivo inactivation studies of either *EZH1*, *EZH2*, *EED*, or *SUZ12* suggest their implication in neuronal maturation and migration, spinal cord development, synaptic plasticity, astrocyte or oligodendrocyte differentiation, and myelination of the central and peripheral nervous systems (reviewed by Liu et al. [31]).. As neurological issues have also been observed in Weaver and other overgrowth syndromes [21], this argues in favour of the implication of *SUZ12* in learning disabilities in *NF1* deletion patients.

Although constitutive depletion of PRC2 core proteins is responsible for embryonic lethality in mice, complete genetic loss- or gain-of-function variants in somatic tissues constitute a driver event for several tumour types through a major alteration of transcription regulation and the alteration of RAS, WNT, and NOTCH signalling [32], and can be at the origin of lymphoid and myeloid malignancies [33], as well as MPNST [34], this later being significantly more prevalent in *NF1*-deleted patients [15]. Hence, mutually exclusive bi-allelic loss-of-function mutations in the PRC2 core proteins SUZ12 and EED are recurrently observed in NF1-associated MPNSTs, while not observed in the pre-cancerous tumours, neurofibromas [35, 36]. It is then reasonable to postulate that constitutive heterozygous deletion of *SUZ12* in *NF1* deletion patient represents a risk factor for MPNST development in NF1 deletion patients.

PRC2 time- and tissue-specific epigenetic programming plays a major role in cell fate and organogenesis. Cardiac cell lineage inactivation of PRC2 components causes major cardiac malformations, eventually leading to neonatal lethality (see Wang et al. for review [37]). Comparable phenomenon is observed by selective inactivation of long noncoding RNAs interacting with PRC2, leading to altered cardiogenic gene transcription: *Braveheart* (*Bvht*) [38], *Fetal-lethal noncoding developmental regulatory RNA* (*Fendrr*) [39], *CARdiac Mesoderm Enhancer-associated Noncoding RNA* (*Carmn*) [40], *cardiac-hypertrophy-associated epigenetic regulator* (*Chaer*) [41], *Ppp1r1b* [42], *human-specific heart brake lncRNA 1* (*HBL1*) [43]. The field is still vast and unexplored, and there is much to be done to understand the role of *SUZ12* in the increased risk for cardiovascular malformations in *NF1*-deleted patients.

#### ATAD5

Constitutional mismatch repair (MMR) deficiency (CMMRD) is a tumour predisposition syndrome that shares some clinical features with NF1, and both syndromes are associated with the occurrence of café-au-lait spots, but also high-grade glioma, acute myeloid leukaemia or rhabdomyosarcoma [44]. ATPase family AAA domain-containing protein 5 (ATAD5) belongs to the proliferation cell nuclear antigen (PCNA) RFC-like (RLC) unloader complex, whose major role is to prevent accumulation of PCNA on chromatin after DNA synthesis [45, 46]. PCNA loading/unloading cycling is essential for proper cell cycle timing and genomic stability. It is presumably implicated in several DNA alterations and repair mechanisms, including double-strand breaks (DSB) repair, gross chromosomal rearrangements, and DNA damage tolerance (DDT) pathway, as well as in sister chromatid cohesion, and chromatin and telomere length maintenance.

PCNA is necessary for the recruitment of MMR proteins to the replication fork. In an in vitro model, inactivation of *elg1*, the yeast homolog for *ATAD5*, led to PCNA over-retention on DNA, and subsequent mutation accumulation mediated by improper recruitment of Msh2-Msh6 and Msh2-Msh3 heterodimers [47].

So far, heterozygous mutations in *ATAD5* have been evidenced in endometrial, breast and ovarian cancers [48, 49]. Type-1 and type-2 deletions at the *NF1* locus constitutively result in *ATAD5* haploinsufficiency (Fig. 1), which most probably constitutes an additional risk factor for genomic instability and tumour emergence.

#### ADAP2

Originally named centaurin-α2 (CENTA2) for its amino acid identity with centaurin-α1, ADAP2 (ARFGAP with Dual Pleckstrin homology domains 2) is present in a wide variety of tissues. It appears to predominantly reside in the cytosol but can have a sustained localisation at the plasma membrane under activation of the PI 3-kinase. It functions as a GTPase activating protein (GAP) for ARF6 (ADP-ribosylation factor 6), a protein implicated in actin cytoskeleton remodelling [50]. ADAP2 is able to bind to microtubules and was suggested to act as microtubule-associated protein (MAP) increasing microtubule stability [51].

*ADAP2* is expressed during key stages of heart formation during embryogenesis. Using a morpholino experiment in zebrafish, Venturin et al. [52]. showed that *adap2* inactivation led to inappropriate heart jogging and heart looping, abnormal ventricular size and atrioventricular valve formation at 2 or 3 days post fertilisation. Incomplete alteration of splicing with an additional morpholino did not cause significant defects, suggesting that complete loss of adap2 would be required for cardiovascular malformations to occur. *ADAP2* might thus play a role in the higher prevalence of cardiovascular malformations in *NF1* deletion patients.

Also, ADAP2 is able to regulate type 1 interferon (IFN1) response during viral infection [53], a signalling pathway that might contribute to neurofibroma formation [54].

#### CRLF3

A publication in 2021 identified *Cytokine receptor-like factor 3* (*CRLF3*) as a candidate gene for autistic traits in *NF1* deleted patients [55]. They showed both a hyperproliferation of neural crest cells and an abnormal dendritic maturation in iPSC-cerebral organoid models derived from type-1 deleted cells. Neurogenic defects were also observed in *CRLF3*-inactivated organoids, pointing out to a specific role of the gene in the proper maturation of neurons, though the precise mechanism underlying such regulation remains to be elucidated.

A more recent study pointed out Crlf3 as a negative regulator of IFN1 and IFN-stimulated genes (ISG) production in *Miichthys miiuy* fish [56]. In their experiments, Crlf3 interacted with TANK-binding kinase 1 (TBK1) and promoted its degradation via ubiquitination, presumably preventing cell response to viral infection.

#### RNF135

Douglas et al. [57] identified in 2007 a truncating mutation or complete deletion of *RNF135* in 5 individuals out of 245 unrelated individuals with overgrowth and learning disabilities, plus one patient with a missense variant in *RNF135*. Though this result was suggestive of a direct implication of RNF135 in overgrowth pathogenesis, Visser et al. [58]. did not identify any pathogenic variant in the gene in a cohort of 160 unrelated individuals with Sotos syndrome, but rather missense, synonymous or intronic variants of unknown significance. In a French cohort of patients with autism, Tastet et al. identified the same type of *RNF135* variants, among which p.Arg115Lys appeared significantly over-represented in their cohort compared to control populations [59]. Still, this variant is reported as homozygous in 22 individuals in the gnomAD v2.1.1 database. The *RNF135* gene encodes an E3 ubiquitin ligase, a pathway previously implicated in autism and intellectual disability. Available data leave the question of whether *RNF135* is indeed responsible for overgrowth and learning difficulties open.

Overexpression of *RNF135* inhibited the in vitro proliferation and invasiveness of SCC25 cells, a model of tongue carcinoma, suggesting a putative role in cancer regulation [60].

#### Other genes of the type-1 deletion interval

Over-expression of Oligodendrocyte-myelin glycoprotein (OMpg) resulted in inhibition of NSC proliferation in a model of neurospheres derived from mesencephalon of rat embryos [61], suggesting a complementarity with the role of neurofibromin in dendritic spine morphology and plasticity [62].

UTP6 Small Subunit Processome Component (UTP6), or Hepatocellular carcinoma antigen 66 (HCA66), is a positive regulator of Apaf-1-dependent apoptosis [63]. As such, its loss represents an additional mechanism by which tumour cells might escape from death in *NF1* deletion patients.

There is still little known about the *COPRS* gene, whose interactions with protein-arginine methyltransferase 5 (PRTM5) and histone H4, and variable expressivity among MPNST cell lines, render its implication in tumoral process uncertain [64].

A few micro RNAs genes are present in the *NF1* type-1 deletion interval: *MIR4733*, *MIR4724*, *MIR193A*, *MIR4725*, and *MIR365B*. miR-193a has well-known tumour suppressor functions, in hepatocellular carcinoma [65], endometrioid endometrial adenocarcinoma [66], non-small-cell lung cancer [67], and breast cancer [68]. Though poorly described in MPNST, miRNAs dysregulation holds a great potential for tumour progression and treatment.

**Table 7:** A summary of purported mechanisms correlating specific gene deletions beyond *NF1* to phenotype in *NF1* deletion syndrome

Phenotype	Gene	Proposed mechanism	Reference
Cardiovascular malformations Includes: Pulmonary stenosis, atrial/ventricular septal defects, valve defects, hypertrophic cardiomyopathy, and patent ductus arteriosus.	*SUZ12*	SUZ12 is known to be deleted within *NF1* deletion syndromes and shown to be expressed during a short period of cardiac morphogenesis within the heart atria. Cardiac cell fate is conditioned by PRC2 recruitment through a variety of lncRNAs, including *Bvht*, *Fendrr*, *Carmn*, *Chaer*, *Ppp1r1b51*, and *HBL1*. Additionally, heterozygous flies with a *Suz12* loss of function mutant allele show impaired expression of various Hox genes (e.g. Ubx and Abd-B) required for appropriate cardiogenesis. Haploinsufficiency of this gene may thus contribute to observable cardiovascular malformation.	Venturin et al. 2005 [69] Wang et al. 2022 [37]
	*ADAP2*	Deletion of *ADAP2* within *NF1* deletion syndrome debilitates its role during fundamental phases of cardiac morphogenesis, resulting in defective heart looping and valvulogenesis.	Venturin et al. 2014 [52]
Higher malignant potential	*UTP6* (*HCA66*)	Shown to selectively modulate Apaf-1-dependent apoptosis, resulting in increased downstream caspase activity following cytochrome c release from the mitochondria. *HCA66* depletion severely impaired apoptosome dependant apoptosis, thus *HCA66* haploinsufficiency has been proposed to render *NF1* deletion patients’ cells less susceptible to apoptosis and more amenable to developing malignancy.	Piddubnyak et al. 2007 [63]
	*ATAD5*	Mice exhibiting *ATAD5* haploinsufficiency display a high magnitude of genomic instability and DNA damage hypersensitivity, with the ATAD5 protein shown to hold a regulatory role in stabilizing stalled DNA replication forks. Somatic mutations within gene have also been identified within sporadic human endometrial tumours as well as breast and ovarian tumour cell lines, thus haploinsufficiency of this purported tumour suppressor gene could contribute to MPNST pathogenesis.	Bell et al. 2011 [48] Kuchenbaecker et al. 2015 [49]
	*SUZ12*	Genetic analysis within MPNSTs commonly identifies bi-allelic inactivation of *SUZ12*, suggesting of a possible tumour suppressor function. This function has been hypothesized to involve the SUZ12 protein’s role within the Polycomb repressive complex 2 (PRC2), which epigenetically regulates genes known to organise cell cycle progression, stem cell self-renewal, cell fate decisions and cellular identity.	De Raedt T et al. 2014 [36]
	*MIR193A* & *MIR365B*	These microRNA genes encode mature miRNAs such as miR193a-3p and miR193a-5p with well-known tumour suppressor functions. These have been demonstrated to exhibit downregulation across a multitude of malignancies from breast cancer cell lines to hepatocellular carcinoma and non-small-cell lung cancer. This observation has yet to be validated within MPNSTs; however, highlighting an avenue for future investigation.	Salvi et al. 2013 [65] Yang et al. 2013 [66] Yu et al. 2015 [67] Tsai et al. 2016 [68]
	*RNF135*	*RNF135* overexpression has been shown to inhibit malignant potential of tongue cancer SCC25 cells, promoting expression of tumour suppressors *PTEN* and *TP53*.	Jin et al. 2016 [60]
	*COPRS*	COPRS is involved in regulation of myogenic differentiation, which may, in haploinsufficiency contribute to oncogenic dysregulated differentiation patterns. Whilst *COPRS* has been shown to be overexpressed in some MPNST tissue samples; this finding is inconsistent across the literature with low expression additionally recognised.	Kehrer-Sawatzki et al. 2017 [64]
Overgrowth in stature	*RNF135*	Genomic analysis of individuals with overgrowth phenotypes of unknown cause has highlighted *RNF135* haploinsufficiency to contribute to phenotypes of overgrowth, facial dysmorphism and possibly learning disability. As *RNF135* is found within the *NF1* deletion region at 17q11 and all three of these phenotypes are readily observable within *NF1* deletion syndrome, *RNF135* has been suggested to underlie this correlation.	Douglas et al. 2007 [57]
	*SUZ12*	A *SUZ12* missense mutation has been identified in a patient exhibiting a Weaver-like syndrome that was associated with overgrowth, thus *SUZ12* has been suggested to contribute to this phenotype in *NF1* deletion syndrome. Additional reports of patients with Imagawa-Mastumoto syndrome due to *SUZ12* pathogenic variants contribute to this hypothesis.	Imagawa et al. 2017 [28] 2023 [29]
Intellectual disability	*OMG*	The encoded OMGp is central to regulation of synaptic plasticity and possibly neurogenesis; dysfunction of which have both been correlated to intellectual disability. Haploinsufficiency may therefore contribute to the significantly lower full scale intelligence quotient observed in patients with deletion syndrome compared to patients with intragenic *NF1* mutations. These may form additive effects with *RNF135* and *NF1* haploinsufficiency. Indeed, the former has been linked to proliferative ability of neural stem cells, whilst neurofibromin is established as an important Ras regulator in interneurons influencing hippocampal-dependent learning.	Martin et al. 2009 [61] Bernardinelli et al. 2014 [70] Oliveira & Yasuda. 2014 [62]
	*RNF135*	Genetic screening of patients with various degrees of learning disabilities or autism spectrum disorder, in association or not with overgrowth, identified several truncating, missense, synonymous or intronic variants in the *RNF135* gene. Comparison with control populations showed a significant over-representation of the p.Arg115Lys variant in the group with autism, with several homozygous patients.	Douglas et al. 2007 [57] Visser et al. 2009 [58] Tastet et al. 2015 [59]
	*SUZ12*	Subunits of PRC2 are expressed in the central nervous system (CNS) and its progenitor cells. They have a pivotal role in the development of central and peripheral nervous systems. As neurological issues have also been observed in Weaver and other overgrowth syndromes, *SUZ12* constitutes another candidate gene for causing intellectual disabilities in *NF1* deleted patients.	Liu et al. 2018 [31] Tatton-Brown et al. 2017 [21]
	*CRLF3*	Human induced pluripotent stem cell (hiPSC)-forebrain cerebral organoid (hCO) models from type-1 deleted patients (“total gene deletion” TGD hCOs), like hCOs with *NF1* intragenic mutations, show neural stem cells (NSC) hyperproliferation compared to non-mutated control hCOs, but they also show abnormal dendritic maturation not found in shorter atypical deletion (aTGD hCOs). A single deleterious *CRLF3* missense mutation (c.1166T > C, p.Leu389Pro) was recurrently identified in NF1 children with higher SRS-2 scores for autism evaluation. *CRLF3*-inactivated hCOs have normal NSC proliferation, but abnormal maturation.	Wegscheid et al. 2021 [55]

All genes listed above have been located within the *NF1* deletion region at 17q11.2 (Fig. 1)

## Discussion

Despite exhibiting complete penetrance, phenotypic manifestations of NF1 may vary substantially. In *NF1* deletion syndrome, the sources of this variation have not been confidently established, although the deletion itself has been proposed to contribute. The deletion length and by extension, the deleted adjacent genes may particularly contribute to the relative severity of *NF1* deletion syndrome versus NF1 caused by intragenic mutations. In addition, due to the deletion mechanism, a subset of patients - particularly type-2 deletion [8] - exhibit mosaicism which itself intrinsically contributes to phenotypic variation.

The primary investigation of this study found no significant novel CNVs within the genomes of the 22 *NF1* deletion patients. It was the first study of its type attempting to explore the presence of modifier CNVs in *NF1* deletion patients. A preferable approach to investigating the existence of modifying loci would have been to examine gene expression for a functional analysis; but we were unable to source and experiment with RNA from these patient samples and therefore had no choice but to carry out an exploratory study into the sourced DNA samples.

A positive correlation was however noted within this study, though not significant after correction for multiple testing, between *NF1* deletion length and learning disability, a clinical manifestation more prevalent in *NF1* deletion patients [1, 71]; the power of this study is however extremely low and must therefore be treated with caution. All type-1 deletion patients for which neurodevelopmental data was available in this study (*n* = 16) exhibited learning disability; whilst all type-2 deletion patients for which neurodevelopmental data was available (*n* = 2) did not exhibit learning disability. Whilst conclusions are difficult to propose with such few patient numbers, a much lower incidence of learning disability could occur in type-2 deletion in comparison to type-1. Similarly, another study by Vogt et al. [72]. also investigated two type-2 deletion patients; neither of which exhibited neurodevelopmental retardation. Other studies with larger numbers of patient did not analyse this comparison. Pasmant et al. [14]. reported a 86% incidence of learning disability in type-1 deletion (*n* = 44) which was higher than the 80% incidence in all *NF1* deletion (*n* = 58).

Establishing such a genotype-phenotype correlation could provide significant clinical benefit, especially in the frame of neurodevelopmental. Indeed, as discussed by Lemay et al. [73], encouraging rapid diagnosis aids in earlier provision of management, advice and support crucial to the patient and their families. This support may take the form of genetic counselling or referral to appropriate services for therapy, education, financial services, respite care, or early intervention support programmes. Indeed, respite care has been found to reduce the incidence of child maltreatment, whilst early intervention programmes before and during school years develop the patient’s language, behavioural, physical and self-management skills, and prepare the child for school [74]. Future study analysing the relative phenotypic incidence between deletion types may therefore provide clinically salient conclusions, perhaps contributing to screening procedures that inform subsequent management.

This potentially increased predisposition of type-1 deletion over type-2 in exhibiting the phenotype of learning disability may find its mechanistic basis in loss of genes such as *LRRC37B* located within the extra 0.2 Mb deleted in type-1 deletion (Fig. 1). It was also proposed that the recognisably less severe clinical phenotype in patients with type-2 *NF1* deletion does not relate to the extent of the deletion but rather may be associated with the frequently observed mosaicism stemming from mitotic NAHR causing type-2 deletion, leading to the presence of normal cells that lack the deletion. Unless mosaicism can be quantified (in the relevant tissues), its presence further complicates translation of genotype-phenotype correlations to the clinical setting.

Several genes included in the type-1 deletion region have been suggested to account for the more severe phenotype observed in this group of patients (Table 7). They are predicted to contribute to cardiovascular malformations (*SUZ12*, *ADAP2*), higher malignant potential (*UTP6*, *ATAD5*, *SUZ12*, *RNF135*, *COPRS*, *MIR193A*, *MIR365B*), overgrowth (*RNF135*, *SUZ12*), intellectual disabilities (*OMG*, *RNF135*, *SUZ12*, *CRLF3*). Altogether, *SUZ12*, which is patently altered in the three main types of *NF1* locus deletions, has emerged as the major candidate for most symptoms over-represented in *NF1*-deleted patients.

Overall, the major drawbacks of this study are the incomplete clinical data, inability to acquire sample RNA to directly assess gene expression and the low power. Another previously conducted study by Riva and collaborators [75] showed no significant association of any phenotype with deletion length; although this study was limited to even less patients (*n* = 10).

Future studies based on exome/whole genome re-sequencing may reveal modifying loci or signalling pathways leading to pharmaceutical targets, begetting further development of management. Further research also based on many rigorously clinically characterised deletion and non-deletion patients may highlight specific SNPs largely associated with a single clinical conglomeration of clinical features. Another important issue will be the phenotypic description. The impact of deleted genes does not fully explain the phenotype, and confounding biases involving other factors modulating the phenotype complicate genotype-phenotype correlations.

In addition, it is important to accurately report the phenotype and differentiate between clinical features, consequences, and complications, as emphasized by Vincent M Riccardi [76]. By distinguishing between different levels of phenotypic description, we can gain a better understanding of the causal genetic factors.

In conclusion, this study did not find any unique CNVs (copy number variations) in NF1 deletion syndrome. However, the study did show a positive correlation between the length of NF1 locus deletion and learning disabilities. These findings will help guide future research into establishing correlations between genotypes and phenotypes.

### Electronic supplementary material

Below is the link to the electronic supplementary material.

Supplementary Material 1

## Data Availability

The datasets used and/or analysed during the current study available from the corresponding author on reasonable request.
