# Supplementary material for: Correlation between large rearrangements and patient phenotypes in NF1 deletion syndrome: an update and review
Source: BMC Med Genomics. 2024 Mar 6;17:73. doi: 10.1186/s12920-024-01843-5 (PMC10919053; doi:10.1186/s12920-024-01843-5)
Supplement: Supplementary file 1 — Supplementary Material 1 [file 12920_2024_1843_MOESM1_ESM.pdf]

Supplementary Table 1. Evaluation of the over-representation of *NF1* deletions larger than 1.2 Mb for each clinical feature, including Patient 2 (deletion >5.5 Mb).

With sample of **Patient 2**		*NF1* **deletion size >1.2 Mb**				
		**No**	**%[1]**	**Yes**	**%[1]**	**p-value[2]**
Overall Severity of NF	Mild/moderate (N=9)	2	22.2	7	77.8	0.544
	Severe (N=13)	1	7.7	12	92.3	
Plexiform neurofibromas	No (N=8)	1	12.5	7	87.5	1.000
	Yes (N=11)	1	9.1	10	90.9	
Lisch Nodules	No (N=4)	0	0	4	100	1.000
	Yes (N=10)	1	10.0	9	90.0	
Macrocephaly	No (N=5)	0	0	5	100	1.000
	Yes (N=10)	1	10.0	9	90.0	
Learning Difficulties	No (N=2)	2	100	0	0	**0.007**
	Yes (N=16)	0	0	16	100	
Scoliosis	No (N=14)	2	14.3	12	85.7	1.000
	Yes (N=5)	0	0	5	100	
Other Malignancies	No (N=17)	2	11.8	15	88.2	1.000
	Yes (N=4)	0	0	4	100	

[1] Percentage calculated over the total of patients for which the data were available (N)

[2] Fisher's exact test

Supplementary Table 2. Evaluation of the over-representation of *NF1* deletions larger than 1.2 Mb for each clinical feature, excluding Patient 2 (deletion >5.5 Mb).

Without sample of **Patient 2**		*NF1* **deletion size >1.2 Mb**				
		**No**	**%[1]**	**Yes**	**%[1]**	**p-value[2]**
Overall Severity of NF	Mild/moderate (N=9)	2	22.2	7	77.8	0.553
	Severe (N=12)	1	8.3	11	91.7	
Plexiform neurofibromas	No (N=7)	1	14.3	6	85.7	1.000
	Yes (N=11)	1	9.1	10	90.9	
Lisch Nodules	No (N=4)	0	0	4	100	1.000
	Yes (N=9)	1	11.1	8	88.9	
Macrocephaly	No (N=5)	0	0	5	100	1.000
	Yes (N=9)	1	11.1	8	88.9	
Learning Difficulties	No (N=2)	2	100	0	0	**0.007**
	Yes (N=15)	0	0	15	100	
Scoliosis	No (N=13)	2	15.4	11	84.6	1.000
	Yes (N=5)	0	0	5	100	
Other Malignancies	No (N=16)	2	12.5	14	87.5	1.000
	Yes (N=4)	0	0	4	100	

[1] Percentage calculated over the total of patients for which the data were available (N)

[2] Fisher's exact test

Supplementary Tables 3. Evaluation of the over-representation of *NF1* deletions larger than 1.35 Mb for each clinical feature, including Patient 2 (deletion >5.5 Mb).

With sample of **Patient 2**		***NF1* deletion size >1.35 Mb**				
		**No**	**%[1]**	**Yes**	**%[1]**	**p-value[2]**
Overall Severity of NF	Mild/moderate (N=9)	5	55.6	4	44.4	0.074
	Severe (N=13)	2	15.4	11	84.6	
Plexiform neurofibromas	No (N=8)	3	37.5	5	62.5	1.000
	Yes (N=11)	3	27.3	8	72.7	
Lisch Nodules	No (N=4)	2	50.0	2	50.0	0.520
	Yes (N=10)	2	20.0	8	80.0	
Macrocephaly	No (N=5)	2	40.0	3	60.0	1.000
	Yes (N=10)	3	30.0	7	70.0	
Learning Difficulties	No (N=2)	2	100	0	0	0.098
	Yes (N=16)	4	25.0	12	75.0	
Scoliosis	No (N=14)	4	28.6	10	71.4	1.000
	Yes (N=5)	1	20.0	4	80.0	
Other Malignancies	No (N=17)	6	35.3	11	64.7	0.281
	Yes (N=4)	0	0	4	100	

[1] Percentage calculated over the total of patients for which the data were available (N)

[2] Fisher's exact test

Supplementary Tables 4. Evaluation of the over-representation of *NF1* deletions larger than 1.35 Mb for each clinical feature, excluding Patient 2 (deletion >5.5 Mb).

Without sample of **Patient 2**		***NF1* deletion size >1.35 Mb**				
		**No**	**%[1]**	**Yes**	**%[1]**	**p-value[2]**
Overall Severity of NF	Mild/moderate (N=9)	5	55.6	4	44.4	0.159
	Severe (N=12)	2	16.7	10	83.3	
Plexiform neurofibromas	No (N=7)	3	42.9	4	57.1	0.627
	Yes (N=11)	3	27.3	8	72.7	
Lisch Nodules	No (N=4)	2	50.0	2	50.0	0.530
	Yes (N=9)	2	22.2	7	77.8	
Macrocephaly	No (N=5)	2	40.0	3	60.0	1.000
	Yes (N=9)	3	33.3	6	66.7	
Learning Difficulties	No (N=2)	2	100	0	0	0.110
	Yes (N=15)	4	26.7	11	73.3	
Scoliosis	No (N=13)	4	30.8	9	69.2	1.000
	Yes (N=5)	1	20.0	4	80.0	
Other Malignancies	No (N=16)	6	37.5	10	62.5	0.267
	Yes (N=4)	0	0	4	100	

[1] Percentage calculated over the total of patients for which the data were available (N)

[2] Fisher's exact test

Supplementary Table 5. Evaluation of the over-representation of *NF1* deletions larger than 1.40 Mb for each clinical feature, including Patient 2 (deletion >5.5 Mb).

With sample of **Patient 2**		***NF1* deletion size >1.40 Mb**				
		**No**	**%[1]**	**Yes**	**%[1]**	**p-value[2]**
Overall Severity of NF	Mild/moderate (N=9)	7	77.8	2	22.2	0.380
	Severe (N=13)	7	53.8	6	46.2	
Plexiform neurofibromas	No (N=8)	5	62.5	3	37.5	1.000
	Yes (N=11)	8	72.7	3	27.3	
Lisch Nodules	No (N=4)	3	75.0	1	25.0	1.000
	Yes (N=10)	7	70.0	3	30.0	
Macrocephaly	No (N=5)	4	80.0	1	20.0	1.000
	Yes (N=10)	7	70.0	3	30.0	
Learning Difficulties	No (N=2)	2	100	0	0	0.529
	Yes (N=16)	10	62.5	6	37.5	
Scoliosis	No (N=14)	8	57.1	6	42.9	1.000
	Yes (N=5)	3	60.0	2	40.0	
Other Malignancies	No (N=17)	11	64.7	6	35.3	0.618
	Yes (N=4)	2	50.0	2	50.0	

[1] Percentage calculated over the total of patients for which the data were available (N)

[2] Fisher's exact test

Supplementary Table 6. Evaluation of the over-representation of *NF1* deletions larger than 1.40 Mb for each clinical feature, excluding Patient 2 (deletion >5.5 Mb).

Without sample of **Patient 2**		***NF1* deletion size >1.40 Mb**				
		**No**	**%[1]**	**Yes**	**%[1]**	**p-value[2]**
Overall Severity of NF	Mild/moderate (N=9)	7	77.8	2	22.2	0.642
	Severe (N=12)	7	58.3	5	41.7	
Plexiform neurofibromas	No (N=7)	5	71.4	2	28.6	1.000
	Yes (N=11)	8	72.7	3	27.3	
Lisch Nodules	No (N=4)	3	75.0	1	25.0	1.000
	Yes (N=9)	7	77.8	2	22.2	
Macrocephaly	No (N=5)	4	80.0	1	20.0	1.000
	Yes (N=9)	7	77.8	2	22.2	
Learning Difficulties	No (N=2)	2	100	0	0	1.000
	Yes (N=15)	10	66.7	5	33.3	
Scoliosis	No (N=13)	8	61.5	5	38.5	1.000
	Yes (N=5)	3	60.0	2	40.0	
Other Malignancies	No (N=16)	11	68.8	5	31.2	0.587

Yes (N=4)	2	50.0	2	50.0

[1] Percentage calculated over the total of patients for which the data were available (N)

[2] Fisher's exact test
